# Autism Symptoms, Executive Functioning and Academic Progress in Higher Education Students

**DOI:** 10.1007/s10803-019-04267-8

**Published:** 2020-01-14

**Authors:** Renée Dijkhuis, Leo de Sonneville, Tim Ziermans, Wouter Staal, Hanna Swaab

**Affiliations:** 1 Department of Clinical Neurodevelopmental Sciences, Leiden University, Leiden, The Netherlands; 2 Leiden Institute for Brain and Cognition, Leiden, The Netherlands; 3 Department of Psychology, University of Amsterdam, Amsterdam, The Netherlands; 4 Karakter Universitair Centrum, Nijmegen, The Netherlands; 5 Radboud Universitair Medisch Centrum, Nijmegen, The Netherlands; 6 Present Address: Department of Brain and Cognition, University of Amsterdam, Amsterdam, The Netherlands

**Keywords:** Higher education, Autism, Executive functioning, Young adults

## Abstract

Many students with autism spectrum disorders (ASDs) attending higher education drop out prematurely. The predictive value of self-reported daily executive functioning (EF) and (cognitive) performance-based EF (mental flexibility and working memory) for academic progress was evaluated in 54 young adults with ASD (M_age_ = 22.5, SD = 2.4, 72% male). Regression analyses showed that autism symptom severity explained 12% of variance in academic progress, which was raised to 36% by adding self-reported daily EF, and to 25% by adding performance-based EF. It is suggested that EF is a candidate marker for academic progress in higher education students with ASD and a candidate target for early intervention.

 According to estimates in the US, the lifetime prevalence of autism spectrum disorder (ASD) ranges between 1.25% based on 2011–2013 data to 2.24% in 2014 (Zablotsky et al. 2015) with 1.70% as the latest estimate (Centers for Disease and Control 2019). Nowadays, individuals with ASD with moderate to high intelligence are likely to follow postsecondary education including college and university programs. Based on findings in the USA it is assumed that between 0.7 and 1.9% of young adults without concurrent intellectual disability meet criteria for autism (White et al. 2011), and numbers are reported to be increasing (Hillier et al. 2018) in the US with 46% since 2000 (Shmulsky et al. 2017). As students in higher education are not required to inform the institute about their diagnosis, exact numbers of students with autism in higher education are not available. While many individuals with ASD are able to cope with the intellectual demands of college, they might possibly struggle with other factors that are critical for academic success; for example limited interpersonal competence, problems with social relationships, problems with executive functioning (EF), poor emotional regulation and comorbid psychopathology, such as high levels of stress and anxiety (Alverson et al. 2019; Glennon 2001; Van Hees et al. 2015; White et al. 2016). Earlier studies indeed demonstrate that students with autism in higher education show an increased incidence of repeating courses and dropping out without a degree in comparison to their typically developing (TD) peers (e.g. White et al. 2011). Also, Mawhood and Howlin (1999) noted that while many children with autism successfully complete mainstream education, their employment levels in adulthood are disappointing. Especially for more intelligent individuals with ASD, it might be extra important to maintain academic progress and to obtain an university diploma as this will facilitate getting a job at a level where they can use their skills and work in an environment where they are amongst like-minded individuals. Many studies have focused on the needs of adolescents and young adults with autism in the transition from high school to higher education, underlining the need of a carefully planned transition, appropriate accommodations, and support (see Wehman et al., 2014, for a review). Concerning postsecondary students with ASD, the majority of research in this domain is only descriptive and interview-based (Anderson and Butt 2017; Gelbar et al. 2014; Longtin 2014). Consequently, current knowledge about the direct relationship between cognitive skills and academic progress in individuals with autism is very limited. Clearly, more research is needed to identify student characteristics that are related to academic progress in young adults with autism.

Most of the research on the relation between student characteristics and academic progress in autism comes from studies with children. In a review on academic success in children with ASD aged 5–18 years, Keen et al. (2016) found almost exclusively studies focusing on IQ and language abilities. They could not conclude on any strong patterns as these studies showed contradicting results, for example between teachers’ and parents’ ratings on symptom reduction in relation to academic achievement (Manti et al. 2011). Some researchers observed that autism severity (Eaves and Ho 1997) and improved social skills (Estes et al. 2011) are related to academic achievement in autism. Overall, youth with autism, even those with higher intelligence, tend to perform poorer with respect to academic results than their TD peers (Ashburner et al. 2008; Troyb et al. 2014). Mayes and Calhoun (2008), found that children with autism display weaknesses in attention, graphomotor, and speed compared to control children. Performance in these areas, belonging to the domain of executive functions (EF), was found to predict academic achievement. These weaknesses have also been found in other children with developmental disorders, like in ADHD. It has been suggested that EF predicts achievement in academic domains over and above general intellectual functioning in typical development (Latzman et al. 2010). EF encompasses a broad range of higher-order cognitive functions supporting abstract reasoning, decision making and social regulation (e.g. cognitive flexibility, inhibition, working memory and planning/organizing), which are necessary for goal-directed behavior. Basic elements of EF (working memory, inhibition, and cognitive flexibility) subserve successful self-regulation (Hofmann et al. 2012) which is clearly necessary for college life, with its emphasis on independence and self-determination. Examples of skills that need optimal EF are; tracking deadlines, time management, keeping class notes and materials organized, coping with schedules that change from day to day and long-term assignments. According to Wolf et al. (2009), planning, organizing and timely completion of assignments are among the most challenging aspects of higher education for students with ASD. For many students with autism, the additional change of living independently when transferring from college to higher education poses extra challenges like keeping up with health, sleep patterns, laundry and meals in addition to their academic and social lives.

It is known that many students with autism experience difficulties in several aspects of EF (Adreon and Durocher 2007; Dijkhuis et al. 2017). Research in children with ASD shows that executive attention is linked to their academic abilities. May et al. (2013) found that attention switching (marker for cognitive flexibility) is associated with both mathematics and reading performance in children with ASD. However, St. John et al. (2018) found that set shifting (cognitive flexibility) at age 6 was related to math achievement, but not to spelling or word reading at age 9 in children with ASD. Assouline et al. (2012) found that in children with high IQ and ASD, working memory is associated with reading and written language. This result was however not replicated by Oswald et al. (2016) as they did not find this relation when IQ and test anxiety were accounted for. Spaniol et al. (2017) found that attention training (CPAT) significantly improved academic performance (maths, reading comprehension and copying text) in children with ASD, showing the importance of attention in many academic areas. Studies of the EF profile of children and adolescents with ASD show that the EF profile is particularly characterized by flexibility and planning deficits as evaluated by performance tasks. But findings are mixed, which is likely due to the differences amongst different age groups and the heterogeneity of the ASD population (Demetriou et al. 2018; Hill 2004; Kenworthy et al. 2008). Studies of EF in adults with ASD employing cognitive performance tasks show EF impairments to be especially related to flexibility, generativity, and spatial working memory. When using informant reports of daily EF problems, clear EF deficits have been found in adults without intellectual disability and ASD (Wallace et al. 2016).

While adults with autism who have been in universities themselves report specific difficulties in daily functioning tasks that place a high emphasis on EF (Robertson and Ne’eman 2008), research investigating the cognitive profile of higher education students with ASD is rather scarce. In a study by Tops et al. (2014) it was found that that differences between young adults with ASD and TD peers appeared almost exclusively on tasks that rely on the integration of different skills. The authors suggested that this originates from problems in cognitive flexibility in ASD. Shmulsky et al. (2017) showed that young adults with autism in postsecondary education who display impaired behavioral regulation (self-reported inhibitory control, shifting, and emotional control) were more likely to earn lower grades than students with autism who reported typical behavioral regulation. To our knowledge, Shmulsky’s paper is the first that explores the relation between study achievement—operationalized as the end-of-year grade point average (GPA)—and EF in higher education students with ASD.

The current study focuses on the question whether EF can help predict academic progress in addition to autism symptom severity in higher education students with ASD. By using not only a self-report measure of EF, but also performance-based measures of EF, we aim to add to the literature in this domain. Different from Shmulsky et al. (2017), we focus on study pace rather than on GPA. We hypothesized that, within the ASD population, problems in EF result in a delay in academic progress, in addition to autism symptom severity.

## Method

### Participants

Fifty-four young adults with ASD (M_age_ = 22.48, SD = 2.43) were recruited for this study, which is part of a study measuring cognitive and behavioral functioning, academic progress and quality of life in higher education students with ASD. All participants were postsecondary students enrolled in higher education in the Netherlands. To increase generalizability, both males and females were included (72% male). The ASD group was recruited through Stumass; a non-profit organization providing services for students with ASD who are enrolled in university programs or universities of higher professional education. Stumass provides guided living homes where students with autism live together, and ambulatory guidance for students that are able to live on their own. In order to be enrolled in Stumass, applicants are required to have received a formal clinical diagnosis of ASD based on the Diagnostic Statistic Manual of Mental Disorders (DSM) criteria (version dependent on what was customary at the time of referral: DSM-III-R/DSM-IV/or DSM-IV-TR), provided according to Dutch protocols. An additional requirement for enrollment in Stumass is that co-morbid psychopathology, if present at entry, is either in remission or of minimal impact on daily functioning of the student. The research protocol was approved by the Medical Ethics Committee of Leiden University Medical Center (NL39057.058.12) and written informed consent was obtained from all participants.

## Measurements

### Autism Symptom Severity

 To evaluate severity of autism symptoms, all participants completed the Dutch self-report version of the Social Responsiveness Scale for Adults (SRS-A; Constantino and Todd 2005). The SRS consists of 65 questions with higher scores indicating more social impairment and more severe ASD traits. The questionnaire comprises the scales social awareness, social communication, social motivation, and autistic mannerisms and gives a total score. A validation study (Constantino et al. 2003) indicated that the SRS was significantly correlated with the ADI-R; with coefficients higher than 0.64. The Dutch version of the SRS has been validated and normed. T scores between 65 and 75 correspond to a ‘mild or moderate’ range of severity, and scores of 76 and higher are in the ‘severe’ range.

### Intelligence

IQ levels were estimated with the V-BD short form of the Dutch version of the Wechsler Adult Intelligence Scale-Fourth Edition, based on the Vocabulary and Block design subtests (WAIS-IV 2008). Total IQ was estimated with the formula [3 × (sum of normed scores) + 40] (Tellegen and Briggs 1967). The V-BD short form is considered a valid estimation of intelligence, it correlates highly with the estimated Full Scale Intelligence Quotient (TIQ) of the WAIS-IV (r = 0.86) (Denney et al. 2015) and has good reliability and validity in both clinical (Denney et al. 2015; Girard et al. 2015) and non-clinical populations (Crawford et al. 2008).

### Academic Progress

In the Netherlands, higher education entails two forms of tertiary education: university education (academic oriented) and higher vocational education (practice oriented). Each curriculum in higher education consists of 60 European Credit Transfer System (ECTS) per year and it has been found that ‘the number of credits earned’ is an appropriate measure for students’ academic progress (Beekhoven et al. 2004; Van Den Berg and Hofman 2005). Each individuals’ ECTS were asked half a year after the initial measurements for autism traits, intelligence and EF. Academic progress is assessed by computing the students’ obtained number of ECTS relative to the total number of ECTS the student could have collected (30 per semester) at the time he/she finished the questionnaire.

### Executive Functioning

As both performance-based and self-reported behavioral measures of executive function provide important information about an individual’s efficiency and success in achieving goals (Toplak et al. 2013), it was decided to use multiple measures to assess EF in this study. The subjective, but ecologically valid self-report version of the Behavior Rating Inventory of Executive Function (BRIEF-A; Roth et al. 2005) was used to obtain information on EF related behavior. Two computerized subtests of the Amsterdam Neuropsychological Tasks (ANTs; De Sonneville 1999, 2014) were used to measure specific cognitive domains of EF.

#### Daily Executive Functioning

The BRIEF-A is a standardized rating scale that assesses the frequency (‘often,’ ‘sometimes,’ or ‘never’) of executive function or self-regulation problems in the everyday environment that have occurred in the last 4 weeks. It is composed of 75 items which are divided over 9 non-overlapping theoretically and empirically derived clinical scales; Inhibit, Shift, Emotional Control, Self-Monitor, Initiate, Working Memory, Plan/Organize, Task Monitor and organization of Materials. See Rabin et al. (2011) for an extensive description of the subscales. The T scores for the subscales, derived from comparisons with normative age groups, are used. Higher scores are indicative of greater perceived impairment in EF and T scores of 65 or higher are categorized as clinically significant. The BRIEF-A has demonstrated reliability, validity, and clinical utility as an ecologically sensitive measure of EF in healthy individuals and also those presenting with a range of psychiatric and neurological conditions (Roth et al. 2005).

#### Performance Executive Functioning

From the *ANT*, the Shifting Attentional Set-Visual (SSV) and the Spatial–Temporal Span (STS) tasks were used. The Shifting attentional Set-Visual (SSV) subtest measures both inhibition and cognitive flexibility. This task consists of three parts in which the participant has to respond to the movement of a square that jumps randomly to the left or right on the screen. In part 1, compatible responding is required: the participant has to follow the movement of the green square (compatible condition—press left/right key on left/right move). In part 2, incompatible responses are required compared to the just trained compatible condition: the square is red and the participant has to move in the opposite direction (incompatible condition), requiring the subject to inhibit the prepotent response. In part 3, a mix of part 1 and 2 trials, cognitive flexibility is required as the participant has to flexibly switch between the two response alternatives, depending on the color of the square. Speed (reaction time, RT) and accuracy (number of errors) are the main outcome parameters. The task model predicts an increase in errors and/or a decrease in speed when inhibition or flexibility is required. Inhibition is operationalized as the difference in performance between parts 1 and 2, cognitive flexibility is operationalized as the difference in performance between part 1 and the compatible trials of part 3, with larger values denoting poorer functioning (slower speed and/or more errors as a result of higher task demands). The STS subtest of the ANT is designed to measure working memory, using squares in a 3 × 3 visual spatial grid. These squares are pointed out by a hand animation in a specific order, with increasing complexity. The test provides two scores: the number of correctly identified squares irrespective of temporal order and the number of squares that are identified in the correct order, which latter condition imposes larger memory demands. The task model predicts that the memory score will be lower when the order criterion is applied. Working memory is operationalized as the difference between these two scores, with a larger value denoting poorer working memory. For a more detailed description of the tasks, including figures, see De Sonneville et al. (2005), (task SSV) and Van Der Meer et al. (2012) and Ziermans et al. (2017) (task STS). Validity coefficients and reliability estimates of the ANT are satisfactory (Günther et al. 2005; De Sonneville 2014). The ANT has been used in various clinical and non-clinical populations, including individuals with ASD (Oerlemans et al. 2013; Van Der Meer et al. 2012; Zmigrod et al. 2013) and individuals with ASD and high IQ (Njiokiktjien et al. 2001; Ziermans et al. 2017).

### Procedure

The assessment of EF was part of an assessment protocol that lasted approximately 3 h in total. The cognitive part (≈ 90 min), including the ANT and the abbreviated WAIS, was always administered first. The ANT was administered on a laptop computer. At the end of the performance session, the participants were debriefed and received a voucher of 20 Euros for their participation in the first part. In addition, they were asked to fill out online questionnaires afterwards. Subsequently, they received an e-mail with a link to the questionnaires so that they could answer the questions at home at their own convenience. Upon returning these questionnaires, they were rewarded with a written report of their cognitive strengths and difficulties in the study. Approximately half a year after the first assessment, the participants received an e-mail with a link to follow-up questionnaires, including information on their academic progress and ECTS at the moment. Students who participated in this second part of the study, received another voucher of 20 Euros for their participation.

### Statistics

All analyses were conducted in IBM SPSS (v.21). All data was checked for normality of the distributions and outliers. Outliers defined as more than 3 standard deviations (z-scores) from the mean were checked for their influence on the data. One outlier on IQ was found, this individual was excluded from further analysis due to extreme performance anxiety during testing that ruined his performance. Also, one individual was excluded from the ANT flexibility measures analysis, and one individual from the ANT inhibition measures analyses, due to extreme scores. One subject did not complete the STS task of the ANT. Level of significance was set at *p* < 0.05.

First, the correlations of age, gender, and IQ with academic progress were calculated as these covariates could potentially influence academic progress. As no significant linear correlations were detected, it was decided not to control for these variables in further analysis. The ANT variables appeared to be skewed which was dealt with by applying a natural log transformation, resulting in acceptable skewness varying between .36 and .69. Academic progress data showed a reasonable skewness with values of − .49. To examine relationships between the variables of interest, Pearson correlation coefficients were computed between academic progress with the SRS-A (total score), the BRIEF-A (subscales- and total score) and the transformed ANT scores (representing the operationalization of inhibition, cognitive flexibility and working memory). Pearson correlations coefficients were identified as weak (0.1–0.3), moderate (0.3–0.5) or strong (> 0.5), according to Cohen (1988).

Next, to answer our research questions, hierarchical multiple regression analyses were performed. Two separate analyses for the self-report and the performance-based EF measures were performed, with the SRS-A total score entered in the first step and those subscales from the EF measures that correlated significantly with academic progress were entered in the second step (all steps Enter method). Level of significance was set at *p* < 0.05. Assumptions for linear regression analysis (normality, linearity, multicollinearity and homogeneity of variance) were met. To provide more robust statistics, subsequent analyses were performed with 1000 resamples bootstrapping with 95% bias corrected and accelerated confidence intervals (CIs).

## Results

The sample characteristics for the remaining participants regarding sex, age, estimated IQ, academic progress and autism symptom severity are given in Table 1. For academic progress, data from 14 participants was missing because they had either stopped their studies (*n* = 8), switched to vocational education (*n* = 4), not attended the follow-up study (*n* = 1), or because they experienced symptoms of depression at the time of the follow-up (*n* = 1). When comparing these fourteen drop outs with the rest of the sample (*n *=39), it was found that they did not significantly differ in terms of IQ, SRS and EF.

**Table 1:** Group characteristics

	ASD (*n* = 53)
Male sex (%)	71.7
Age in years, *M* (*SD*)	22.5 (2.4)
WAIS-IV Total IQ, *M* (*SD*)	118.28 (11.22)
SRS-A Total score^a^, *Mdn* (range)	63.00 (48–94)
% ECTS obtained^b^	66.40 (28.40)

^a^T-score; missing data (*n* = 2)

^b^Missing data (*n* = 14)

### Correlations with Academic Progress

Correlational analyses with academic progress were performed with 39 participants for the SRS-A, 37 for the BRIEF-A, and 38 for the *ANT.* A significant medium correlation with academic progress emerged for the total score of the SRS-A (*r* = − .35, *p* = .033). Significant correlations with academic progress also emerged for some scales of the BRIEF-A, ordered from strong to moderate correlations: plan/organize (*r* = − .57, *p* < .001), initiate (*r* = − .49, *p* = .002), total score (*r* = − .45, *p* = .005), task monitor (*r* = − .40, *p* = .014) and working memory (*r* = − .38, *p* = .021). These negative correlations indicate that a higher autism score and poorer daily EF were associated with poorer academic progress. Correlational analysis showed moderate correlations between academic progress and *ANT* scores; speed (*r* = − .34, *p* = .036) and accuracy (*r* = − .33, *p* = .046) of cognitive flexibility, and working memory (*r* = − .35, *p* = .033), indicating that poorer cognitive flexibility and working memory were associated with poorer academic progress.

### Predicting Academic Progress in Autism with Daily EF

 Initial regression analysis with the four subscales of the BRIEF-A entered in the second step resulted in a significant model with none of the predictors being significant (*p*_*range*_= .21–.69). It was therefore decided to use only the subscale Plan/Organize from the BRIEF-A in the final regression model, as this subscale correlated highest with academic progress. The final model is shown in Table 2. The first model with SRS-A total score was significant [*F*(1, 35) = 4.94, *p* = .033], explaining 12% of the variance in academic progress; the second model adding the subscale plan/organize of the BRIEF-A showed a statistically significant improvement [*F*(2, 34) = 9.38, *p* = .001], and tripled the explained variance in academic progress to 36%. In this last model, only plan/organize remained a significant predictor of academic progress (β = − .51, *p* = .001). The bootstrapping results showed that in model 1, autism symptom severity was significantly predictive of academic progress (*B* = − 1.01, *p* = .038, 95% bootstrap CI = [− 1.94; − .11]). In model 2, autism symptom severity no longer appeared significantly predictive of academic progress (*B* = − .56, *p* = .17, 95% bootstrap CI = [− .29; .24]), while Plan/organize was significantly predictive of academic progress (*B* = − 1.15, *p* = .004, 95% bootstrap CI = [− 1.79; − .35]).

**Table 2:** Summary of multiple linear regression analysis predicting academic progress by the Behavior Rating Inventory of Executive Functioning Adult version (BRIEF-A)

Predictor variables	*R*	*R*^2^	*R*^2^*change*	Outcome measure (academic progress)				*p* value
				*B*	*SE B*	β	*F* change	
Model 1	.35	.12					4.94	.033*
Constant				132.08	29.91			
SRS-A Total score				− 1.01	.45	− .35		
Model 2	.60	.36	.24				12.23	.001*
Constant				180.93	29.54			
SRS-A Total score				− .56	.42	− .20		.184
BRIEF-A Plan/Organize				− 1.15	.33	− .51		.001*

*p < .05

### Predicting Academic Progress in Autism with Performance EF

Because of a substantial collinearity between the two flexibility subscales of the ANT (accuracy and speed), it was decided to delete flexibility speed from the list of predictors. Initial regression analysis with the remaining subscales of the ANT that correlated significantly with academic progress showed that the model with the subscales cognitive flexibility accuracy and working memory was best in explaining academic progress. The model for predicting academic progress from cognitive EF tasks is shown in Table 3. The second model, after adding the cognitive flexibility accuracy and working memory measures from the *ANT*, was statistically significant [*F*(3, 32) = 3.63, *p* = .023] and the explained variance in academic progress increased from 12 to 25%. However, in this last model, none of the predictors remained a significant predictor of academic success. The bootstrapping results showed that in model 1, autism symptom severity was significantly predictive of academic progress (*B* = − .99, *p* = .046, 95% bootstrap CI = [− 1.91; − .08]). In model 2, autism symptom severity no longer appeared significantly predictive of academic progress (*B* = − .60, *p* = .22, 95% bootstrap CI = [− 1.82; .30]), this was also found for cognitive flexibility accuracy (*B* = − 35.88, *p* = .075 95% bootstrap CI = [− 72.8; 5.04]) and working memory (*B* = − 7.74, *p* = .15, 95% bootstrap CI = [− 17.10; 5.04]). To explore the individual contribution of the two EF predictors to the explained variance, a post hoc three steps multiple regression analysis was conducted. Adding cognitive flexibility to the model raised the explained variance of 12.2% with 7.8% to 20%. By adding working memory in the third step the explained variance increased with 5.4% to 25.4%.

**Table 3:** Summary of the linear regression analysis predicting academic progress by subscales of the Amsterdam Neuropsychological Tasks (ANTs)

Predictor variables	*R*	*R*^2^	*R*^2^*change*	Outcome measure (academic progress)				*p* value
				*B*	*SE B*	β	*F* change	
Model 1	.35	.122					4.72	.037*
Constant				131.75	30.25			
SRS-A Total score				− .99	.46	− .35		.037*
Model 2	.50	.254	.13				3.63	.023*
Constant				206.74	52.81			
SRS-A Total score				− .65	.46	− .23		.173
Cognitive flexibility accuracy				− 35.82	20.99	− .27		.098
Working memory				− 7.8	5.11	− .25		.137

*p < .05

## Discussion

This study aimed to test whether EF can help to predict academic progress in higher education students with ASDs, in addition to severity of autism symptoms. In line with our expectations, it was found that when taking autism into account, self-reported daily EF contributes to the prediction of academic progress, with self-reported abilities of planning and organizing being the best predictor of academic progress in young adults with ASD. Better developed planning and organizing skills are associated with stronger academic progress, raising the explained variance in academic progress from 12% (ASD symptoms only) to 36% (both symptoms and planning/organizing skills). We therefore conclude that, in addition to the severity of autism symptoms, self-reported daily EF is a valuable predictor of academic progress in young adults with ASD. Better performance during tasks that ask for cognitive flexibility and working memory appeared to be related to stronger academic progress as has been demonstrated by the correlational analysis. Together, severity of autism symptoms, cognitive flexibility and working memory performance are significantly predictive of academic progress. This was shown by a significant model in regression analyses, raising the explained variance in academic progress from 12% (ASD symptoms only) to 25% (both symptoms and cognitive performance). However, none of the three predictors in the final model was significant in itself, which is why these results should be interpreted with caution. Nevertheless, referring to the 95% CI from the bootstrap analysis, the point estimates of *B* are compatible with the idea that poorer performance-based EF is associated with poorer academic progress. For decisions about whether to pursue a research idea further, we concur with Amrhein and Greenland (2019) and colleagues that there is no simple connection between a *p* value and the probable result of subsequent studies. The resulting model is informative, corroborates with the literature, and may therefore serve as a basis for further exploration.

Problems in planning/organizing reflect impairments that are often reported by clinicians working with children with high IQs and ASD (American Psychatric Association 2013; Wolf et al. 2009) and the current findings add to the growing evidence suggesting that EF deficits are of relevance in autism in adulthood (Wallace et al. 2016). Also, the finding that problems in cognitive flexibility and working memory are related to academic progress is in line with previous studies in both children (Assouline et al. 2012; May et al. 2013; St. John et al. 2018) and adults with ASD (Tops et al. 2014; Shmulsky et al. 2017). Previous research showed more consistent EF deficits in autism when a self-report method (the BRIEF) was used than when performance EF tasks were used (Rosenthal et al. 2013; Van Eylen et al. 2015; Wallace et al. 2011). This might be explained by the fact that cognitive EF tasks, like the subtasks of the *ANT* used in the present study, are designed to assess specific, strictly operationalized, aspects of EF under relatively optimal conditions. Also, with performance tasks, the test/tester is ignorant of the individual level of academic progress, which underlines the strength of this study measuring EF in different ways.

One might question whether or not the prediction of GPA versus academic progress is more successful. Looking at the GPA at the time of the follow-up in the current study, it was found that number of credits earned and GPA are strongly correlated (*r *= .59, *p* < .001). This result indicates that those students who earned more credits, also had a higher GPA. This seems in line with the Dutch higher education system, in which all studies provide a curriculum of 60 ECTS per year. Not many students take extra courses, and it is not expected of them. Furthermore, in the Netherlands there are no scholarships based on GPA, leading us to assume that GPA is not as important in the Netherlands as it is in other countries. As the correlation of the possible predictors with GPA were lower than with academic progress we did not pursue this issue any further.

Some limitations of our study need to be mentioned. First, academic progress can be measured not only in grades, but also in, for example, increased learning, increased independence and self-determination and positive social experiences. The current study looks at a more global academic progress and these factors were not included in the current study. Also, the current sample consists of higher education students with ASD with mean IQ levels in the high average range, so these results cannot be generalized to autism samples with lower level IQs. However, as these findings are similar to patterns of difficulties found with respect to flexibility, planning and organization in both children and adolescents with autism without intellectual disability (Granader et al. 2014), the current literature on EF difficulties in autism suggests that the importance of EF in predicting study outcome can be extended to autism samples with other IQ- and age ranges. Finally, the number of participants in the regression analyses in relation to the number of predictors should be preferably larger than realized in the current analyses. The rather low number of participants in the performance EF regression analysis may also be one of the reasons why none of the predictors in the regression analysis was significant. Nevertheless, significant correlations of EF skills with academic progress were found, indicating a specific relation between (poor) planning/organizing, working memory, cognitive flexibility and (lower) academic progress in higher education students with ASD.

To our knowledge, this is the first study exploring *both* cognitive performance-based and self-report measures of EF as potential predictors of academic progress in young adults with autism in higher education. Where Shmulsky et al. (2017) found poorer self-reported EF related to lower GPA, we found that poorer EF, both self-reported *and* performance-based, resulted in a slower study pace. The results in this study reflect the impact of everyday metacognitive demands that students encounter in higher education. Higher education settings place huge demands on their students’ flexibility as schedules which appear to be “set”, frequently change. In line with Shmulsky et al. (2017), we suggest that caregivers or tutors and students who are planning for college should assess EF to identify strengths and areas of concern, which can also be shared with relevant disability service offers, counselors or consultants in higher education, to maximize successful transition. Just like in the Stepped Transition in Education Program for Students with ASD (STEPS; White et al. 2017) EF can possibly be improved by teaching effective problem-solving and goal-setting skills. Additionally, according to Hillier et al. (2018), support groups with fellow ASD students, consisting of weekly meetings addressing common challenges experienced by students in university settings, enhance success for students with autism. The participants reported for example an increase in behaviors like implementing strategies to reduce stress and anxiety and learning how to set and meet appropriate goals. Also, they reported improved EF skills, academic-related skills, understanding of how to access resources and supports on campus, and social understanding. We recommend that students get the chance to improve their self-regulation while still in high school or while preparing for higher education, for example by learning how to deal with unexpected blocks of time and schedule changes. Many academic scholars with ASD routinely use computers, mobile phones, and organizational and planning tools to manage their schedules and workloads, which has been confirmed by Robertson and Ne’eman (2008). They suggest that the logical and systematic aspects of computers and other information technologies strongly appeal to the cognitive processing strengths in rule-based, logical, and systematic thinking possessed by many people with ASD. Therefore, next to using these tools, computer-based programs to improve EF not only in children but also in adults with ASD seem promising. Indeed, positive results of EF training have already been reported. For example the executive function training ‘Unstuck and On Target’ seems a promising intervention for improving planning/organizing, flexibility and problem-solving skills in children with autism, as a recent paper shows improvements in these areas due to EF training, even more than when compared to a social skills intervention (Kenworthy et al. 2014). This may potentially be an avenue worth pursuing as improved flexibility may impact other EFs like working memory, planning and organizational skills. Latzman et al. (2010) found in a population sample that cognitive flexibility influences many areas of academic functioning (e.g. reading and science) while inhibition, another executive function, is especially important for math in adolescence. This stresses the importance of different EFs for different academic areas. The evaluation of personalized EF training in individuals with ASD and further research into relevant domains of cognitive functioning that might be targets for intervention, could lead to better self-regulation skills and improved outcomes in individuals with ASD.

### Conclusions

The current study shows that EF potentially plays an important role in academic progress for young adults with ASD. Future research should aim at replicating these findings across different countries and samples of students with ASD, and disentangle the contribution of EF to academic progress compared to other relevant predictors in ASD like IQ and comorbidity. These findings emphasize that clinicians working with children, adolescents and adults with ASD in education settings, should be aware of the impact that EF has on academic progress. Enhancing EF in ASD might not only enhance academic progress, and therefore the chances of graduating, but also overall quality of life and outcome for this group. We urge the field to highly prioritize systematic research into mechanisms related to study drop-out and the development of interventions to promote successful studying for students with ASD.
